# Evaluation of a new point-of-care oral anti-HCV test for screening of hepatitis C virus infection

**DOI:** 10.1186/s12985-020-1293-7

**Published:** 2020-01-31

**Authors:** Lili Liu, Mingyuan Zhang, Lei Hang, Fei Kong, Hongqing Yan, Yumei Zhang, Xiangwei Feng, Yuanda Gao, Chang Wang, Heming Ma, Xu Liu, Mengru Zhan, Yu Pan, Hongqin Xu, Junqi Niu

**Affiliations:** Department of Hepatology, The First Hospital of Jilin University, Jilin University, Changchun, 130021 China

**Keywords:** Hepatitis C virus, Oral fluid, Point-of-care, OraQuick, HCV screening

## Abstract

**Background:**

Hepatitis C virus (HCV) infection is a public health issue for which an effective universal screening method is urgently needed. An oral anti-HCV test could provide a noninvasive and rapid screening strategy for HCV infection. This study evaluated the performance of a new point-of-care oral assay developed by Well for the detection of HCV antibody.

**Methods:**

Individuals from three centers with and without HCV infection were enrolled. All participants were tested for oral HCV antibody using the Well assay and for serum HCV antibody using established tests (ARCHITECT i2000 anti-HCV assay and InTec serum anti-HCV assay). For participants who obtained positive results, HCV RNA was tested for verification. Some patients underwent the OraQuick HCV test at the same time, and some self-tested with the Well assay during the same period.

**Results:**

A total of 1179 participants, including 486 patients with chronic HCV infection, 108 patients with other liver diseases, and 585 individuals who underwent physical examination, were enrolled. The Well anti-HCV test had a sensitivity of 91.88% (95% confidence interval [CI]: 88.97–94.09%) and a specificity of 98.00% (96.58–98.86%) for oral HCV antibody detection. The consistency between the Well and InTec assays was 97.02% (1138/1179). The consistency between the Well and OraQuick assays was 98.50% (197/200). Furthermore, the results of self-testing were highly consistent with those of researcher-administered tests (Kappa = 0.979). In addition, the HCV RNA results also showed that HCV RNA could only be detected on 1 of the 39 false-negative samples, and for 172 positive HCV RNA results, 171 could be detected by the Well oral anti-HCV assay.

**Conclusions:**

The Well oral anti-HCV test offers high sensitivity and specificity and performed comparably to both the OraQuick assay and InTec assay for HCV diagnosis. Thus, the Well test represents a new tool for universal HCV screening to identify infected patients, particularly in regions with limited medical resources.

## Background

Hepatitis C virus (HCV) infection is highly prevalent and a growing public health problem worldwide, especially in low- and middle-income areas. According to the World Health Organization (WHO), the prevalence of HCV infection ranges from 0.5–2.3%, and approximately 71 million people had chronic HCV infection in 2016. Chronic HCV infection remains the leading cause of liver fibrosis, cirrhosis, and even hepatocellular carcinoma, and as such, it is still associated with significant morbidity and mortality [1].

HCV-infected individuals develop an immune response to the virus, but the virus changes during replication and escapes, leaving 55–85% of patients with chronic infection [1]. However, among HCV-infected patients who are at an elevated risk of progressive liver disease, less than 5% are aware of their HCV infection [2]. The rate of undiagnosed HCV infection is still high because cases are often asymptomatic or only mildly symptomatic. In most instances, HCV carriers are diagnosed only once they develop abnormal liver function or symptoms of cirrhosis [3]. Consequently, these patients do not receive treatment early enough to prevent life-threatening complications, and accordingly, the disease burden related to HCV infection is expected to further increase in the future.

At present, the main treatment for HCV infection is interferon-free direct-acting antiviral (DAA) therapy, which has been shown to be very effective and tolerable as well as associated with fewer adverse events than previous treatments [4]. The advent of oral DAAs, which provide a cure rate of more than 90%, has brought about a new era of treatment and made the elimination of HCV possible [5–7]. Considering the social and economic burden of HCV infection as well as the high cure rates achieved with DAA drugs, in 2016, the WHO proposed a strategy to eliminate viral hepatitis infection, aiming to control both viral hepatitis B and C infection by 2030 [8]. The identification of undiagnosed infected people is a key challenge in reaching this goal. In 2017, the WHO provided the first guidelines on testing for chronic HCV infection, recommending strategies for testing, including risk-based screening, general population screening, and birth cohort screening. However, the current HCV screening rates are still low in many developing countries, possibly due to limited hepatitis testing facilities, a lack of effective testing policies or national guidelines, expensive testing methods, and complex diagnostic algorithms [9]. Moreover, based on the 2017 World Bank list of economies, approximately 80% of HCV infections worldwide are in low- and lower-to-middle-income areas [10].

HCV screening is often performed using an enzyme-linked immunosorbent assay (ELISA) or chemiluminescence immunoassay (CLIA) for HCV antibody detection [11]. However, these tests are time-consuming and require large, specialized equipment and trained technicians, making them expensive and difficult to apply in areas with poor medical resources [12]. In contrast, point-of-care tests are simple and rapid, with easily understood results, and thus, such tests for HCV infection can provide opportunities to broaden screening efforts within nontraditional settings [13]. Moreover, tests for antibodies in oral fluid are relatively noninvasive, cost-effective, more convenient and safer than those requiring serum collection and testing [14]. For these reasons, point-of-care tests have been developed for the detection of antibodies in oral fluids, including the OraQuick® HCV rapid antibody test designed for HCV infection screening [15]. Testing of the OraQuick test in Europe, the United States, South Korea, and other regions has demonstrated its high sensitivity and specificity for HCV antibody detection [16–18]. Unfortunately, however, the assay is expensive, making widespread application in areas with limited resources difficult.

The Well oral anti-HCV assay is a new test developed by Jiangsu Well Biotech Co., Ltd. that is designed to detect antibodies against HCV in oral fluids with the advantages of low cost and simplicity.

The present study provides the first evaluation of the diagnostic accuracy of the Well oral anti-HCV assay for the purpose of determining its potential utility for HCV screening in areas with limited medical resources.

## Materials and methods

### Study design

This multicenter study was conducted in three hospitals: the Sixth People’s Hospital of Zhengzhou (Center 1), the Sixth People’s Hospital of Shenyang (Center 2), and the First Hospital of Jilin University (Center 3). The study participants included HCV-infected patients, non-HCV-infected liver disease patients, and healthy subjects. The study was approved by the institutional review committees of all three centers, and all participants signed an informed consent form.

Oral fluid samples from all participants were tested for HCV antibody using the Well oral anti-HCV assay, and blood samples were collected for serum HCV antibody detection as reference data. For participants who agreed, the OraQuick oral anti-HCV assay was also applied. Some participants also enrolled in the self-test subgroup in which they self-tested using the Well oral anti-HCV assay 30 min after the researcher-administered testing. Furthermore, HCV RNA detection was performed for samples with positive anti-HCV results (Abbott assay and Well assay).

To minimize bias, the study used a blinded method; i.e., blood sample testing, oral sample testing and results interpretation were performed by different researchers unaware of the results of the other tests.

### Inclusion criteria

The following inclusion criteria were applied for participant enrollment. Individuals were at least 18 years of age and consented to participate in the study by signing the informed consent form. Additionally, individuals who were enrolled in the self-test subgroup had to be able to understand the test procedure.

### Data and sample collection

Demographic and clinical data were collected using a questionnaire that gathered information on age, sex, medical history, medical education background, etc.

**Fig. 1:**
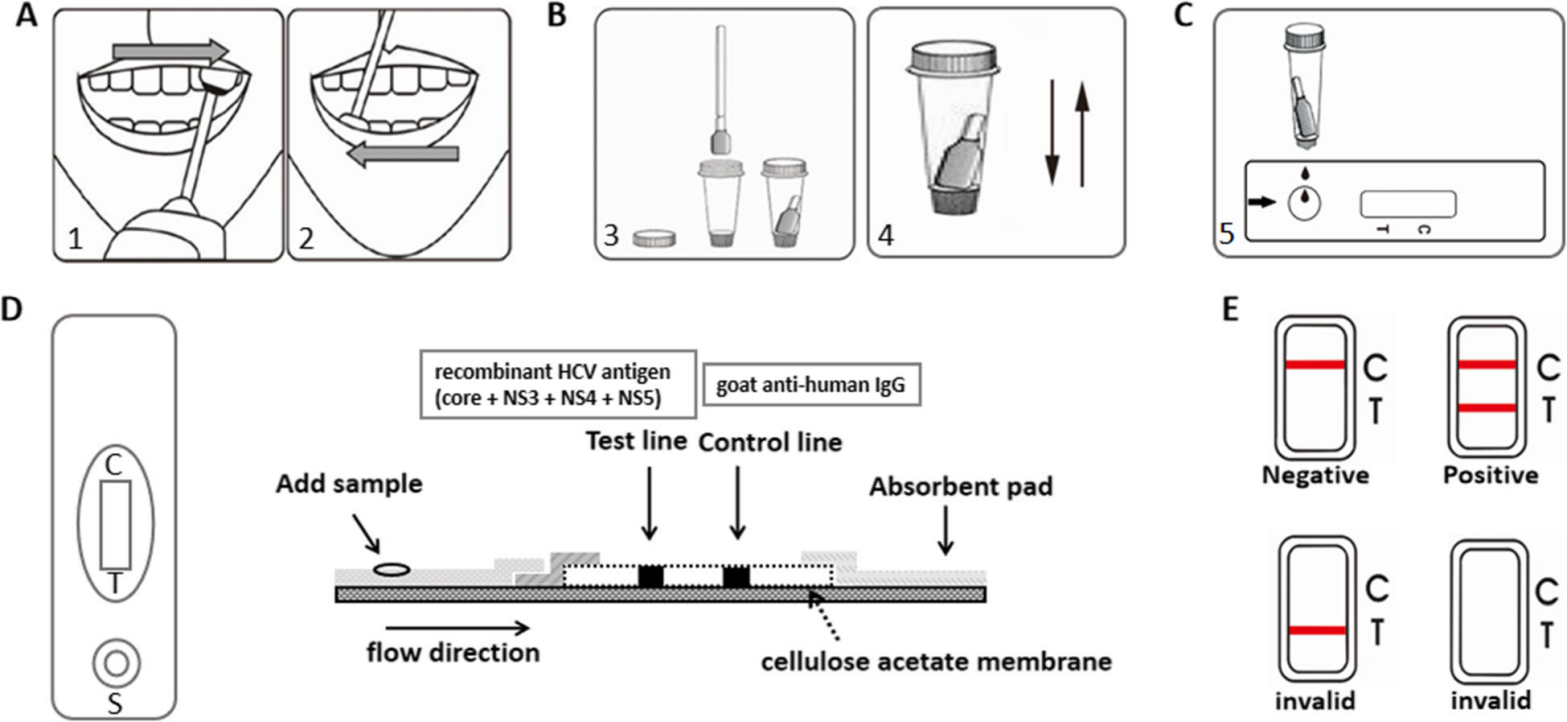
Schematic and procedures of the Well anti-HCV assay. **a** Oral sample collection; **b** mixing of oral liquid with diluted buffer solution and storage in collection bottle; **c** addition of oral samples for testing; **d** schematic of the shell and diagram illustrating the working principle of the internal test strip. HCV antibody first binds to the recombinant protein G conjugated with colloidal gold to form a complex. Under the action of chromatography, the complex binds to the recombinant HCV antigen (core + nonstructural [NS]3 + NS4 + NS5), which was immobilized on the detection area (test-line); and **e** results interpretation

Oral fluid samples were collected by swabbing of the area between the buccal mucosa and gingiva in the order shown in Fig. 1a. The swab was then dipped into diluted buffer solution in the sample storage bottle and mixed thoroughly by squeezing (Fig. 1b). These oral fluid samples were used for Well assay detection directly and do not require long-term preservation. For subjects who need to collect oral fluid samples more than once, the time interval should exceed 30 min. Venous blood samples were collected during the same visit as that of the oral fluid samples. After centrifugation, the serum was separated and stored at − 80 °C. All serum samples were transported to the central laboratory of the Department of Hepatology, First Hospital of Jilin University for testing.

### Well assay and reference measurement methods

The Well anti-HCV assay (Jiangsu Well Biotech Co., Ltd., Jiangsu, China) utilizes an indirect immunoassay method in a lateral flow device to identify HCV antibody. The assay is contained within a white shell, and a test strip with a gold colloid is located inside the shell (Fig. 1d). Oral fluid is added to the sample hole by squeezing the collection bottle (Fig. 1c). When there is HCV antibody in the specimen, it first binds to the recombinant protein G conjugated with colloidal gold to form a complex. Under the action of chromatography, the complex binds to the recombinant HCV antigen (core + nonstructural [NS]3 + NS4 + NS5) which was immobilized on the detection area (Test-line). Approximately 15–20 min after the oral fluid sample is loaded into the device, a visible reddish test line that indicates the positive detection of HCV antibody in the sample will appear if the sample is positive. Whether the result is negative or positive, the area of control line should appear red. If no red control line appears, the result is invalid (Fig. 1e).

For reference measurements, serum HCV antibody was detected using an automated third-generation CLIA (i2000; Abbott Diagnostics, Chicago, IL, USA) to obtain reference standards. A serum HCV antibody level greater than or equal to 1 signal-to-cut-off ratio (S/Co) was defined as positive. The InTec serum anti-HCV assay (InTec Products, Inc., Xiamen, China), a point-of-care assay based on the colloidal gold principle, was also used to obtain auxiliary reference measurements. The InTec assay used serum samples for HCV antibody screening, and its operating method and working principle are similar to those of the Well assay. The OraQuick oral anti-HCV assay (OraSure Technologies, Inc., Bethlehem, PA, USA) was also applied in some participants as auxiliary reference measurement. It has features similar to those of the Well assay, but the interpretation time is 20–30 min. An HCV RNA assay (Haoyuan Co., Ltd., Shanghai, China) with a limit of detection of 100 IU/ml was used to measure the serum levels of HCV RNA.

The testing of all serum samples (including with the Abbott anti-HCV assay, InTec anti-HCV assay, and HCV RNA assay) was completed in the central laboratory. After professional training, qualified researchers conducted the testing in strict accordance with the standards and instructions, and two reference standards were tested and quality control were carried out before the test every day.

### Statistical analysis

The diagnostic performance of the Well assay was evaluated using the serum HCV antibody measurement results as the reference. The sensitivity, specificity, accuracy, positive predictive value (PPV), negative predictive value (NPV), positive coincidence rate and negative coincidence rate as well as their 95% confidence intervals (CIs) were calculated. The Kappa value was determined to assess the extent of consistency, with a Kappa value of > 0.75 indicating high consistency and a Kappa value of < 0.4 indicating weak consistency. Statistical analyses were performed using GraphPad Prism version 5.0 (GraphPad, Inc., San Diego, CA, USA). Statistical significance was defined as *P* < 0.05.

## Results

### Demographic and clinical characteristics of the study participants

A total of 1179 participants were enrolled in the three centers, including 351 in Center 1, 345 in Center 2, and 483 in Center 3. Overall, 550 (46.6%) participants were male, and 629 (53.4%) were female. The median age of all participants was 50 years. All participants were divided into three groups according to their primary disease history: 486 in the HCV infection group, 108 in the non-HCV-related liver disease group, and 585 in the healthy control group. The general demographic and clinical data of the study participants are presented in Table 1.

**Table 1:** General demographic and clinical characteristics of the study participants

Characteristic	Center 1	Center 2	Center 3	Total
Age, median (range)	49(28,57)	51(44,58)	50(39,57)	50(37,57)
Sex, male (n, %)	150(42.7%)	162(47.0%)	238(49.3%)	550(46.6%)
Diagnosis, n				
HCV infection	112	106	268	486
Non-HCV-related liver diseases	28	55	25	108
HBV infection	28	43	19	90
Non-HBV-related liver disease	0	12	6	18
Healthy control participants	211	184	190	585
Total	351	345	483	1179

*Abbreviations*: *n* Number, *HCV* Hepatitis C virus, *HBV* Hepatitis B virus

### Clinical performance of the well oral anti-HCV assay

HCV screening was performed for 1179 individuals using the Well oral anti-HCV assay as well as the Abbott serum assay. The results of serum HCV antibody detection served as the reference standard. The findings of HCV antibody detection were inconsistent between the Well assay and the serum assay in 53 cases. Therefore, the sensitivity of the Well oral anti-HCV assay in the present study was 91.88% (95% CI 88.97–94.09%), and its clinical specificity was 98.00% (95% CI 96.58–98.86%). Additionally, the overall accuracy was 95.50% (95% CI, 94.16–96.56%; Table 2).

**Table 2:** Performance of the Well assay according to the reference results of the Abbott assay

Well oral anti-HCV assay	Serum anti-HCV (Abbott assay) (*n* = 1179)								
	Center 1 (*n* = 351)		Center 2 (*n* = 345)		Center 3 (*n* = 483)		Centers 1–3		Total
	Positive	Negative	Positive	Negative	Positive	Negative	Positive	Negative	
Positive, n	109	4	91	8	241	2	441	14	455
Negative, n	3	235	12	234	24	216	39	685	724
Total	112	239	103	242	265	218	480	699	1179
Sensitivity^a^ (%) (95% CI)	97.32(91.79,99.31)		88.35(80.16,93.57)		90.94(86.66,93.99)		91.88(88.97,94.09)		
Specificity^a^ (%) (95% CI)	98.33(95.48,99.46)		96.69(93.35,98.45)		99.08(96.37,99.84)		98.00(96.58,98.86)		
PPV^a^ (%) (95% CI)	96.46(90.65,98.86)		91.92(84.24,96.19)		99.18(96.74,99.86)		96.92(94.77,98.24)		
NPV^a^ (%) (95% CI)	98.74(96.06,99.67)		95.12(91.42,97.34)		90.00(85.31,93.36)		94.61(92.64,96.09)		
Accuracy^a^ (%) (95% CI)	98.01(95.86,99.11)		94.20(91.17,96.26)		94.62(92.20,96.33)		95.50(94.16,96.56)		
Kappa value^a^	0.954		0.860		0.892		0.906		

Note: ^a^Sensitivity, specificity, PPV, NPV, accuracy and Kappa value were calculated based on serum HCV antibody test results detected using the Abbott assay. *n* Number, *PPV* Positive predictive value, *NPV* Negative predictive value, *95%CI* 95% confidence interval

### Clinical performance of the well oral anti-HCV assay according to the InTec assay

A total of 1173 individuals were tested for HCV using the Well oral anti-HCV assay and the InTec serum assay. The other 6 participants were not tested with the InTec assay due to insufficient serum samples. The results of serum HCV antibody detection performed by the InTec assay were used as the reference standard. The sensitivity and specificity of the Well oral anti-HCV assay in the present study were 95.42% (95% CI 92.98–97.08%) and 98.04% (95% CI 96.65–98.88%), respectively. Additionally, the overall consistency was 97.02% (95% CI, 95.87–97.86%; Table 3).

**Table 3:** Performance of the Well assay according to the reference results of the InTec assay

Well oral anti-HCV assay	Serum anti-HCV (InTec assay) (*n* = 1173)								
	Center 1 (*n* = 347)		Center 2 (*n* = 343)		Center 3 (*n* = 483)		Centers 1–3		Total
	Positive	Negative	Positive	Negative	Positive	Negative	Positive	Negative	
Positive, n	106	4	92	7	240	3	438	14	452
Negative, n	3	234	5	239	13	227	21	700	721
Total	109	238	97	246	253	230	459	714	1173
Positive coincidence rate^b^ (%) (95% CI)	97.25(91.57,99.29)		94.85(87.82,98.09)		94.86(91.17,97.12)		95.42(92.98,97.08)		
Negative coincidence rate^b^ (%) (95% CI)	98.32(95.47,99.46)		97.15(93.98,98.75)		98.70(95.92,99.66)		98.04(96.65,98.88)		
Consistency^b^ (%) (95% CI)	97.98(95.81,99.10)		96.50(93.92,98.05)		96.69(94.65,97.99)		97.02(95.87,97.86)		
Kappa value^b^	0.953		0.914		0.934		0.937		

Note: ^b^Positive coincidence rate, negative coincidence rate, consistency and Kappa value were calculated based on serum HCV antibody test result detected using the InTec assay. *n* Number, *95%CI* 95% confidence interval

### Consistency between the results of the well oral anti-HCV assay and the OraQuick anti-HCV assay

The OraQuick assay was additionally applied for a few participants in each of the three centers. The OraQuick assay showed good performance for detecting HCV antibody, with a sensitivity of 90.00% (95% CI 80.73–95.27%) and a specificity of 98.33% (95% CI 93.51–99.71%). The accuracy was 95.00% (190/200). Overall, consistent findings were obtained with the Well oral ant-HCV assay and the OraQuick assay for 98.50% of the cases, with a Kappa value of 0.968. Of the three centers, the consistency rate was highest among participants from Center 3, reaching up to 98.55% (Table 4).

**Table 4:** Consistency between the results of the Well oral anti-HCV assay and the OraQuick anti-HCV assay

Well oral anti-HCV assay	OraQuick anti-HCV assay (*n* = 200)								
	Center 1 (*n* = 65)		Center 2 (*n* = 66)		Center 3 (*n* = 69)		Centers 1–3 (*n* = 200)		Total
	Positive	Negative	Positive	Negative	Positive	Negative	Positive	Negative	
Positive, n	24	0	27	1	22	1	73	2	75
Negative, n	1	40	0	38	0	46	1	124	125
Total	25	40	27	39	22	47	74	126	200
Consistency ^c^(%) (95% CI)	98.46(91.00,100.00)		98.48(91.12,100.00)		98.55(91.48,100.00)		98.50(95.48,99.69)		
Kappa value^c^	0.967		0.969		9.67		0.968		

Note: ^c^Consistency and Kappa value were calculated according to the results of the OraQuick assay. *n* Number, *95%CI* 95% confidence interval

### Consistency between the results of self-administered versus researcher-administered well oral anti-HCV tests

The self-test subgroup consisted of 199 participants. The consistency rate between the self-test results and the researcher-administered test results was high, with a Kappa value of 0.979. Inconsistent results were obtained for only 2 cases (Table 5). Notably, according to the anti-HCV serostatus as the reference standard, the results of the researcher-administered tests were correct for one patient, while the results of the self-administered tests were correct for the other patient. The consistency between the Well oral anti-HCV assay with self-testing and that with researcher administration was 98.99% (95% CI 96.17–99.96%).

**Table 5:** Comparison of the consistency of the Well oral anti-HCV test conducted by researchers versus participants

Well oral anti-HCV assay, self-testing	Well anti-HCV assay, researcher administered (*n* = 199)								
	Center 1 (*n* = 65)		Center 2 (*n* = 66)		Center 3 (*n* = 68)		Centers 1–3 (*n* = 199)		Total
	Positive	Negative	Positive	Negative	Positive	Negative	Positive	Negative	
Positive, n	24	1	28	0	23	1	75	2	77
Negative, n	0	40	0	38	0	44	0	122	122
Total	24	41	28	38	23	45	75	124	199
Consistency^d^ (%) (95% CI)	98.46(91.00,100.00)		100.00(93.41,100.00)		98.53(91.37,100.00)		98.99(96.17,99.96)		
Kappa value^d^	0.967		1.000		0.967		0.979		

Note: ^d^Consistency and Kappa value were calculated according to the results of the Well oral anti-HCV test conducted by researchers. Consistency means the overall coincidence rate between the Well oral anti-HCV test conducted by researchers and conducted by participants. *n* Number, *95%CI* x95% confidence interval

### False negative results obtained with the well oral anti-HCV assay

**Fig. 2:**
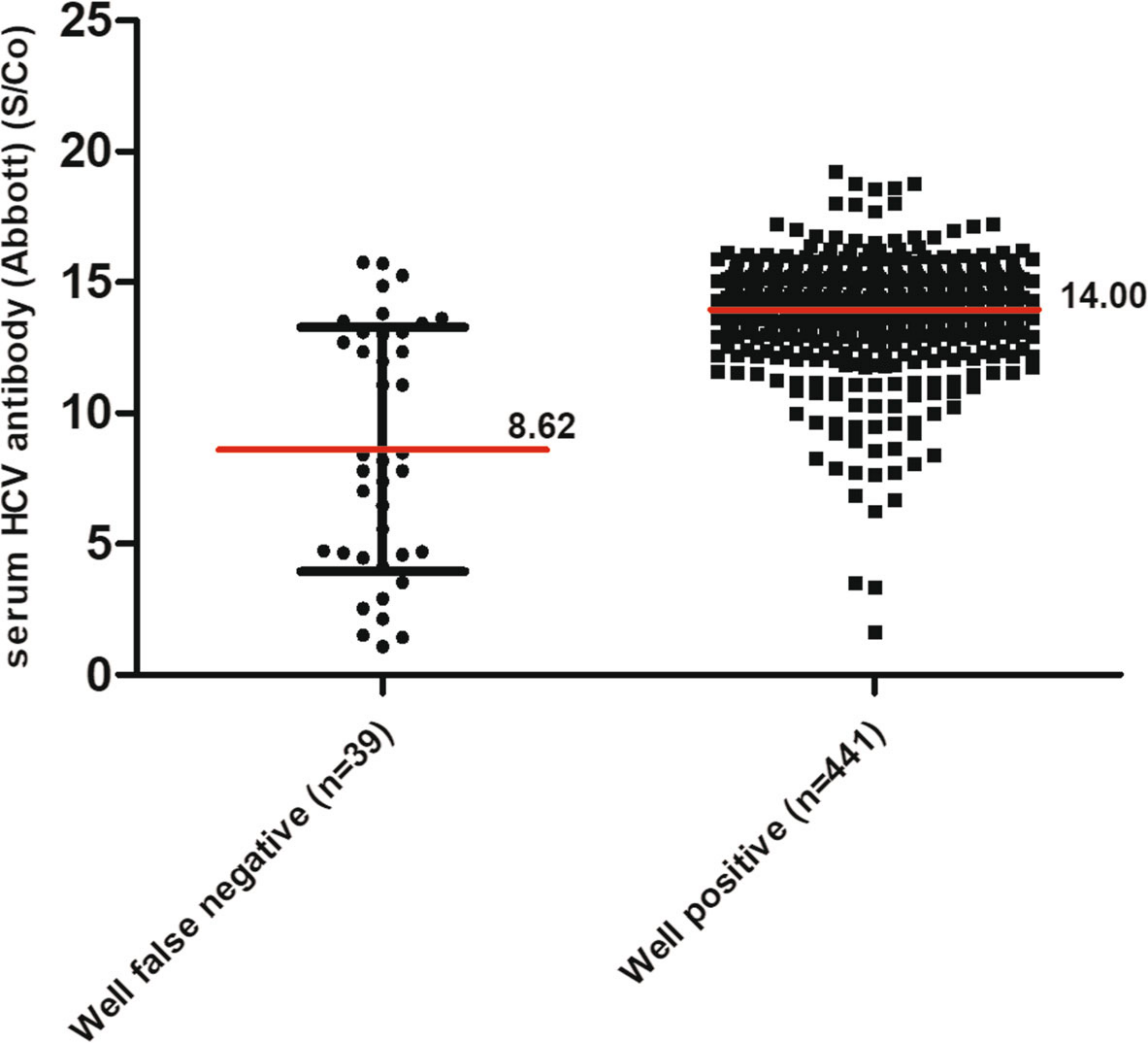
Comparison of serum HCV antibody titers in patients with false negative versus positive results. The False negative and positive results were obtained by the Well assay according to the Abbott assay. Serum HCV antibody titers (S/Co) were obtained by the Abbott anti-HCV assay

Relative to the reference results for serum HCV antibody detection using the Abbott assay, inconsistent results were obtained for a total of 53 cases. For the development of an effective screening tool for HCV, we are more concerned about missed cases than false positive results. Therefore, the results for the 39 cases with a false negative finding were further analyzed. Notably, the false negative rates for each center were 2.68, 11.65 and 9.06%, respectively. Among the 39 cases with false-negative results, 19 cases did not receive treatment due to the absence of treatment indications. One patient had not started treatment after the initial diagnosis. The remaining 19 subjects had received interferon or DAA treatment and achieved a sustained virological response (SVR). The HCV RNA results also showed that HCV RNA could only be detected on 1 of the 39 false-negative samples. In addition, the InTec results showed more than half (21/39) false negative results, with the majority (20/21) of subjects showing low serum antibody titers on the Abbott assay (Table 6). We next assessed whether a false-negative result on oral fluid testing was associated with the serum HCV antibody titer. Comparing the serum HCV antibody titers obtained by the Abbott assay in HCV patients with false-negative Well oral anti-HCV results versus patients with positive Well oral anti-HCV results, it was found that the HCV antibody titers in patients in which the oral HCV antibody was detected correctly were significantly higher than in those with false-negative oral HCV antibody results (14.00 S/Co vs 8.62 S/Co, *P* < 0.001) (Fig. 2).

**Table 6:** Summary of test results for patients with false negative results on the Well assay (*n* = 39)

Subject ID	Well oral anti-HCV assay	Serum anti-HCV (Abbott assay) (S/Co)	OraQuick anti-HCV assay^e^	Serum anti-HCV (InTec assay)	HCV RNA (IU/mL)	Antiviral treatment
30139	–	1.08	NA	–	< 100	no therapeutic indication
30215	–	1.43	NA	–	< 100	no therapeutic indication
30186	–	1.5	NA	–	< 100	no therapeutic indication
30140	–	2.13	NA	–	< 100	no therapeutic indication
20031	–	2.53	–	–	< 100	no therapeutic indication
20122	–	2.9	NA	–	< 100	PEG-IFN and ribavirin, SVR
20019	–	3.53	–	–	< 100	no therapeutic indication
30168	–	4.12	NA	–	< 100	no therapeutic indication
30228	–	4.47	NA	–	< 100	no therapeutic indication
20038	–	4.59	–	–	< 100	no therapeutic indication
30150	–	4.65	NA	–	< 100	no therapeutic indication
20199	–	4.69	NA	–	< 100	no therapeutic indication
30155	–	4.74	NA	–	< 100	no therapeutic indication
30214	–	5.57	NA	–	< 100	no therapeutic indication
30020	–	6.46	–	–	< 100	sofosbuvir/velpatasvir, SVR
30195	–	7.03	–	–	< 100	no therapeutic indication
30317	–	7.41	NA	+	< 100	sofosbuvir/daclatasvir, SVR
30321	–	7.8	NA	+	< 100	no therapeutic indication
20112	–	7.81	NA	–	< 100	PEG-IFN and ribavirin, SVR
30108	–	8.2	NA	–	< 100	PEG-IFN and ribavirin, SVR
30226	–	8.43	NA	–	< 100	no therapeutic indication
30238	–	8.49	NA	–	< 100	PEG-IFN and ribavirin, SVR
20010	–	11.07	–	+	< 100	sofosbuvir/velpatasvir, SVR
30256	–	11.07	NA	+	< 100	PEG-IFN and ribavirin, SVR
30245	–	11.97	NA	+	< 100	PEG-IFN and ribavirin, SVR
20056	–	12.35	NA	–	< 100	no therapeutic indication
30241	–	12.35	NA	+	< 100	PEG-IFN and ribavirin, SVR
30162	–	12.69	NA	+	< 100	no therapeutic indication
10287	–	12.98	NA	+	< 100	sofosbuvir and ribavirin, SVR
10294	–	13.09	NA	+	< 100	sofosbuvir and ribavirin, SVR
20095	–	13.1	NA	+	< 100	PEG-IFN and ribavirin, SVR
30128	–	13.42	NA	+	< 100	sofosbuvir and ribavirin, SVR
30251	–	13.51	NA	+	< 100	PEG-IFN and ribavirin, SVR
20301	–	13.61	NA	+	< 100	PEG-IFN and ribavirin, SVR
30249	–	13.81	NA	+	< 100	PEG-IFN and ribavirin, SVR
20125	–	14.87	NA	+	< 100	elbasvir/grazoprevir, SVR
30246	–	15.25	NA	+	< 100	PEG-IFN and ribavirin, SVR
10025	–	15.72	+	+	< 100	no therapeutic indication
20140	–	15.76	NA	+	6.38E+ 05	treatment naive

Note: Subject ID were assigned according to the order in which subjects participated in the study. The ID consists of five digits, with the first digit representing the center. ^e^7 of the 39 subjects with false-negative results were tested with OraQuick assay. *NA* Not applicable, *PEG-IFN* Pegylated-interferon, *SVR* Sustained virological response

### Ability of the well oral anti-HCV assay to identify HCV RNA positive patients

A total of 495 samples were tested for HCV RNA based on positive anti-HCV results obtained by both Abbott assay and Well assay. Overall, 172 subjects had positive HCV RNA results, and 171 of them had positive results on the Well oral anti-HCV assay.

## Discussion

HCV infection continues to be a global epidemic, creating considerable social and economic burdens. With major breakthroughs in the treatment of HCV infection, the elimination of HCV infection has become realizable. Despite progress in laboratory testing methods for HCV infection, a large number of HCV-infected patients remain undetected, making the eradication of the disease less feasible. Therefore, an effective screening tool for identifying these patients is needed. Due to their simple operation and avoidance of the need for specialized instruments, point-of-care tests can be more easily applied within primary medical institutions and even homes, and as such, they represent a powerful potential means to achieve universal HCV screening. Additionally, since it was determined that HCV antibody can be detected in oral fluid [19], which offers the advantages of noninvasive and easy collection, tests that use oral fluid samples have become desirable. However, the good clinical performance of a test is essential for its widespread adoption. In the present multicenter study, we evaluated the diagnostic performance of a new point-of-care test, the Well oral anti-HCV assay, for HCV antibody detection in oral fluid samples.

Within 15 to 20 min real time, the Well oral anti-HCV assay achieves the rapid detection of HCV antibody with high consistency relative to the serum detection of HCV antibody. The clinical sensitivity was 91.88%, and the NPV was 94.61%. The sensitivity achieved by the Well anti-HCV assay was moderate compared to the values reported in other clinical evaluation studies [20, 21]. The Well assay also showed a high specificity of 98.00% and a high PPV of 96.92% in the present study, with the specificity of the assay in Center 3 reaching 99.08%. Moreover, when the colloidal gold assay (InTec) was used as a auxiliary reference, the consistency rate was 97.02%. These results demonstrate the good performance of the Well oral anti-HCV assay for HCV screening.

In 2010, the Food and Drug Administration approved the OraQuick assay as the first rapid blood test for HCV antibody detection in individuals 15 years and older. Clinical studies with the OraQuick test showed its excellent sensitivity and specificity compared to blood reference tests. In 2017, a meta-analysis of eight studies conducted in the United States, Brazil, South Korea, and Saudi Arabia was conducted to evaluate the diagnostic accuracy of the OraQuick test, and the pooled sensitivity and specificity values were 98 and 100%, respectively [22]. An additional study in Spain found that the clinical sensitivity and specificity of the OraQuick HCV rapid test were 89.9 and 100%, respectively [18]. In the present study, the sensitivity of the OraQuick assay for HCV antibody was 90%, while the specificity was 98.33%. To our knowledge, this is the first time that the accuracy of the assay has been reported in a Chinese population. These slight variations in the sensitivity of the assay may indicate that HCV antibody concentrations in oral fluids can vary among different races. The almost complete concordance between the Well oral anti-HCV assay and the OraQuick assay revealed that their efficacies were highly consistent in our study population. Overall, these results suggest that the Well oral anti-HCV assay is a suitable assay for HCV antibody detection in oral fluid.

The current study also included a subgroup of participants who performed self-testing using the Well oral anti-HCV assay. This subgroup included HCV-infected patients, non-HCV-infected liver disease patients, and healthy individuals. The self-testing results showed high consistency with the results obtained in researcher-administered tests. Of the two inconsistent results, the researchers and participants obtained one correct result each. Notably, no significant differences were found in the sensitivities and specificities of the Well oral anti-HCV test administered by the researchers versus the participants. Recently, Kimble et al. evaluated the performance of the OraQuick HCV assay with self-collected oral fluid and found that the assay showed good performance on self-testing only when the results were interpreted by trained staff [23]. Our findings show that the Well oral anti-HCV assay is user-friendly and could potentially be used for self-testing by untrained users. Such an application could be valuable in a universal HCV screening scheme expandable to primary care as well as home care.

The Well anti-HCV test gave false-negative results in 39 of 480 individuals with an anti-HCV positive serostatus as well as 14 false-positive results in individuals with a negative serostatus. For use as a screening tool for HCV, we are most concerned with the sensitivity of the assay, and therefore, the false-negative results were further analyzed. The HCV RNA results showed that most of the 39 patients with false-negative results on oral testing had no detectable virus in their serum. According to their responses on the medical history questionnaire, half (19/39) of those infected with HCV had received interferon or DAA treatment and had achieved viral clearance, and 19 patients with HCV infection who had not received treatment but had negative serum HCV RNA were considered to have spontaneous viral clearance. Furthermore, we found that the serum HCV antibody titers of the 39 individuals were significantly lower than those of the other patients. About half of these cases generated a low S/Co level of anti-HCV antibodies (1.00 S/Co - 8.00 S/Co). It is well established that patients with spontaneous viral clearance or a SVR have lower levels of antibodies [24]. Additionally, the HCV antibody titer appears to continue to decrease after treatment [25], and 18–34% of HCV-infected patients experience spontaneous clearance after acute infection [26]. Of the 39 patients, 7 were willing to be tested with OraQuick, and only 1 tested positive. The serum InTec results showed that the false negative rate was also higher for samples with a low S/Co on the Abbott results. The waning of humoral responses after treatment-induced viral clearance or spontaneously resolved infection may cause the low amount of antibody in the oral fluid [18] and the false negative results on the Well assay. Based on the above analysis, the high false-negative rate may be related to the presence of a certain number of patients with low S/Co values who are HCV antibody-seropositive but HCV RNA-negative (due to spontaneous viral clearance or treatment-induced clearance), especially in center 2 and center 3. Moreover, the HCV RNA results showed that for 172 cases with positive HCV RNA results, 171 cases were identified by the Well oral anti-HCV assay. This possibility is supported by a study that showed that the sensitivity and specificity were significantly greater when HCV RNA measurement was considered as the reference standard rather than serum HCV antibody detection [27]. Similarly, a previous study found nonreactive results in oral fluid consistent with resolved infection, which had the absence of viremia [17]. Our findings suggest that the Well oral anti-HCV assay is more sensitive for identifying active HCV infections, which indicates that it is better at finding infected patients who require antiviral treatment and may reduce unnecessary medical care for patients who have been cured or for whom the virus has spontaneously cleared.

Importantly, the advantages of the Well oral anti-HCV assay, including rapid and noninvasive testing, simple and equipment-free operation, easy interpretation of results, and low price, are likely to also improve individual compliance and reduce the risk of exposure to blood-borne pathogens. With its good sensitivity and specificity as well as these other important advantages, the Well oral anti-HCV assay satisfies several important criteria for the consideration of HCV screening [28].

The strengths of the present study include the enrollment of participants from multiple centers, the use of multiple methods for obtaining reference standards, and the first evaluation of the performance of the OraQuick in a Chinese population. However, this study also has some limitations. The group with other liver diseases should include patients with other types of conditions, such as HIV infection. Moreover, only a small number of people participated in OraQuick testing.

## Conclusions

The novel Well oral anti-HCV assay can accurately detect HCV antibodies in oral fluid, providing a rapid, inexpensive, equipment-free and user-friendly test for detecting HCV infection. Therefore, it is particularly suitable for large-scale HCV screening in resource-constrained regions as well as for self-testing at home. Such possibilities could expand screening efforts, reducing the future burden of disease related to unidentified HCV-infected patients and supporting attempts to eliminate HCV infection.

## Data Availability

The datasets are available from the corresponding author on reasonable request.

## Abbreviations

anti-HCV: HCV antibody
CI: Confidence interval
CLIA: Chemiluminescence immunoassay
DAA: Direct-acting antiviral
ELISA: Enzyme-linked immunosorbent assay
HCV: Hepatitis C virus
NA: Not applicable
NPV: Negative predictive value
NS: Nonstructural
PPV: Positive predictive value
S/Co: Signal-to-cut-off ratio
SVR: Sustained virological response
WHO: World Health Organization
